# 

**Published:** 1964-07

**Authors:** 


BRISTOL MEDICO-CHIRURGICAL JOURNAL

*(Incorporating the Medical Journal of the South-West)*

Established 1883

VOL. 79 (iii) July 1964 NO. 293

### Contents

RUMINATIONS ON THE TRIGEMINAL NERVE *V. Lambert* 75

TRANSPLACENTAL FOETAL BLOOD LOSS: A COMMON EVENT . . . . *I. D. Fraser* 80

TRICKS OF THE PSYCHOTHERAPIST'S TRADE *D. Russell Davis* 83

POLYNEURITIS COMPLICATING HERPES ZOSTER *E. S. Etuk et al.* 91

PRIMARY SYSTEMIC AMYLOIDOSIS *Clinico-Pathological Conference* 93

REPORTS FROM SOCIETIES . . . . . . . 99

OBITUARY . . . . . . . . . . 100

LOCAL MEDICAL NOTES . . . . . . . 101

*PUBLISHED QUARTERLY*

*ANNUAL SUBSCRIPTION* 16/- *POST FREE*

PUBLISHED UNDER THE AUSPICES OF THE
BRISTOL MEDICO-CHIRURGICAL SOCIETY

### *Editorial Committee*

Dr. N. J. Brown, Southmead Hospital, Bristol. *Joint Editor.*
Dr. A. B. Raper, Department of Pathology, Bristol Royal Infirmary. *Joint Editor.*
Dr. J. Macrae, Ham Green Hospital.
Dr. R. C. Wofinden, Central Clinic, Bristol.
Mr. A. E. S. Roberts, Medical Library, University of Bristol. *Secretary.*

*Local Correspondents*

*Bath* . . Mr. A. D. Bateman, 3 The Circus, Bath.
*Exeter* . . Dr. L. N. Jackson, Mount Jocelyn, Crediton, Devon.
*Gloucester* . Dr. R. F. Jarrett, 9 College Green, Gloucester.
*Plymouth* . Dr. C. R. Croft, Lexden, Hartley Av., Plymouth.
*Taunton* . Dr. M. McAlley, 1 The Crescent, Taunton.
*Torquay* . Dr. A. Robinson Thomas, 20 Devon Square, Newton Abbot, Devon.
*Truro* . . Dr. F. D. M. Hocking, St. Andrews, Perranporth, Cornwall.
*Yeovil* . . Mr. J. A. Vere Nicoll, Knapp House, Yeovil, Somerset.

### NOTICE TO CONTRIBUTORS

Original articles are invited, provided they have not been published elsewhere. Notes of forthcoming events, meetings held, degrees conferred, staff appointments, and other local news are welcomed. The editorial committee reserves the right to make literary corrections.

Illustrations should always be supplied ready for reproduction. All re-touchings or other operations required will be charged to the author. Line drawings should be drawn in Indian ink. We regret that we must ask authors to pay for making blocks, if this is necessary.

J. W. ARROWSMITH LTD., WINTERSTOKE ROAD
BRISTOL.

### *Reports from Societies*

### SOUTH-WEST PAEDIATRICIANS

A meeting of the South West Paediatric Club took place at the Children's Hospital, Bristol, on 7th and 8th December 1963. It was noticeable that paediatricians from other parts of the country, who at some time had worked in the south west, took pleasure in returning to Bristol. Clinical cases were shown and discussed, and several papers were read.

Dr. A. E. Tinkler recorded that the complete and permanent eradication of congenital syphilis had very nearly been accomplished in this country. Nowadays the diagnostic problem in infancy was more commonly serological than clinical. Some of the serological problems presented by fully treated, untreated, and inadequately treated mothers and their children were considered, with particular reference to infants offered for adoption.

Dr. J. S. Cornes described lymphoid polyps and hyperplasia in the intestine. Examination of solitary lymph follicles and Peyer's patches in 129 specimens of small and large intestine showed that the patches increased in size and number from early foetal life until puberty, and did not disappear in old age. They were most noticeable in well-nourished children and in patients with adrenocortical insufficiency. Hyperplasia of these patches in the conical ileocaecal valve of infancy was occasionally the cause of an ileocolic intussusception. Hyperplasia of solitary follicles might present as localized polyps in the rectum, and biopsies from them might be mistaken for lymphosarcoma, with unfortunate results. In certain cases of leukaemia and lymphosarcoma the patches and follicles were greatly enlarged throughout the entire gastrointestinal tract before lymphoid tissue in other parts of the body was affected.

### SYMPOSIUM ON GENETICS AND CHROMOSONE EXAMINATION IN BRISTOL

Dr. V. J. Marrian gave a summary of experiences in the Bristol Genetic Clinic during the 3-year period October 1960 to October 1963, comparing these with those of the previous 6 years described by Dr. Oppé (this Journal, Vol. 77 p. 75). As well as presenting several pedigrees in detail, she made observations on the psychological and social implications of the work, stressing the difficulty of communication to the patient, and the risks of misinterpretation by patient and parents.

Dr. A. B. Raper dealt with a series of routine chromosome cultures performed for diagnostic purposes. 105 patients had been studied, 61 of them children, and all by leucocyte culture; there was a wide range of initial diagnoses. Abnormal karyotypes were found only in mongolism, Klinefelter's syndrome (3/7), Turner's syndrome (2/5), and testicular feminization; 14 out of 15 males with cryptorchidism had normal karyotypes, but 1 was an XX/XXY mosaic. Of 11 children thought to be mongoloid, only 1 was normal; 1 had a D/G translocation. In view of the large number of normal results, and the time employed in furnishing clinicians with this evidence, he thought it was questionable whether a routine "free-for-all" service was worth while.

Dr. F. J. W. Lewis presented, with illustrations, 3 rare examples of chromosome abnormalities:—

1. An XO/X-isochrome X mosaic; a spastic quadriplegic infant demonstrated by Professor A. V. Neale in the Clinical Section of the meeting. The evidence for the presumptive isochrome of the long arm of X was based on morphology, statistical analysis, autoradiography, and the demonstration of abnormal "drumsticks" in some of the polymorph leucocytes.

2. Deletion of the short arm of an autosome of the 17-18 group (Denver) found repeatedly in a mentally deficient male with multiple congenital abnormalities.

3. Presumptive trisomy of 16 (Denver) found in blood and skin cultures from a mentally deficient female with multiple congenital abnormalities.

Mr. M. B. Wingate said that genetic studies on plants and animals had demonstrated the existence of lethal factors responsible for abnormal development or death of the organism at various stages of development. Sometimes their presence could be detected by examination of the chromosomes, and in patients who were "habitual aborters", he and Mrs. Wingate had found translocations to be present in a high proportion of cases. Three out of five couples so far investigated had shown aberrations; two a translocation of the short arms of one chromosome of the 16/18 group to the short arms of a chromosome of the 21/22 group. It was reasonable to assume that translocation of chromosome material might play a part in the aetiology of recurrent foetal loss.

### *Local Medical Notes*

The University of Manchester conferred on Mr. J. R. Nicholson-Lailey the honorary LL.D. on the occasion of the annual conference of the B.M.A.

Dr. M. A. Voyce has been awarded a James Smellie bursary for 1964.
*F.R.C.P.;* F. T. Page and J. A. Cosh.

Obituary: B. E. McConnell.

*Printed by*

J. W. Arrowsmith Ltd., Bristol

